# Ossifying metaplasia of urothelial metastases: original case with review of the literature

**DOI:** 10.1186/s12880-015-0072-1

**Published:** 2015-08-12

**Authors:** Sana Boudabbous, Daniel Arditi, Emilie Paulin, Thibaud Koessler, Anne Laure Rougemont, Xavier Montet

**Affiliations:** Geneva University Hospital, Radiology department, Rue Gabrielle-Perret-Gentil 4, Genève 4, 1211 Switzerland

**Keywords:** Osseous metaplasia, Urothelial tumor, Metastasis, Sarcoma, Percutaneous biopsy

## Abstract

**Background:**

Ossifying metaplasia is an unusual feature of urothelial carcinoma, with only a few cases reported. The largest series included 17 cases and was published in 1991. The mechanism of ossification is unknown and hypotheses of osteogenic precursor cells, inducing bone formation, are proposed.

**Case presentation:**

A 75 year-old patient was treated for a high grade transitional cell carcinoma of the bladder by surgery, chemotherapy and radiotherapy. Histology showed foci of bone metaplasia, both at the periphery of the tumor, and in a lymph node metastasis. 1 year later, a heterotopic bone formation was discovered in the right retroperitoneal space, near the lumbar spine, increasing rapidly in size during follow-up. Several imaging exams were performed (2 CT, 1 MRI, 1 Pet-CT), but in the absence of typical features of sarcoma, diagnosis remained unclear.

Histology of a CT-guided percutaneous biopsy showed urothelial carcinoma and mature lamellar bone. Integration of these findings with the radiological description of extraosseous localization was consistent with a diagnosis of osseous metaplasia of an urothelial carcinoma metastasis. The absence of bone atypia in both the primary and metastases argues against sarcomatoid urothelial carcinoma with osteosarcomatous differentiation.

**Conclusion:**

Osseous metaplasia of an urothelial carcinoma metastasis is unusual, and difficult to distinguish from radiotherapy induced sarcoma, or from sarcomatoid carcinoma. Rapid progression, sheathing of adjacent structures such as vessels (like inferior vena cava in our case) and nerves and bony feature of lymph node metastases necessitate histological confirmation and rapid treatment. Our case illustrates this disease and evaluates the imaging features. In addition we discuss the differential diagnosis of osseous retroperitoneal masses.

## Background

Osseous metaplasia is rarely observed in the stroma of bladder carcinoma, either in the primary tumor [1] or in a metastasis. In an article published in 1991 by Robert H., 17 cases were reviewed [2], with only a few more cases reported since then [3]. This additional finding in itself is considered benign; the main challenge is to differentiate osseous metaplasia from sarcomatoid carcinoma with osteosarcomatous differentiation or from radiation induced osteosarcoma, that would obviously change both the course of the disease and the treatment [2, 4].

Although the mechanisms are still hypothetical, osteogenic precursor cells, a favorable microenvironment, and ossification-inducing stimuli are prerequisites to the heterotopic formation of bone. The hypothesis is that a clone of tumor cells produces bone foci by inducing osteogenesis.

Imaging revealed an osseous mass in the retroperitoneal space, infiltrating posterior structures including vessels and nerves and generating compression of adjacent tissues with bone invasion. We report the case of an osseous metaplasia in urothelial carcinoma, both in the primary and in metastases. This case is illustrative of the course of the disease with its imaging features (which are not specific), and allows discussion of the main differential diagnoses, particularly sarcoma and other rare ossifying pathologies.

## Case presentation

**Fig. 1:**
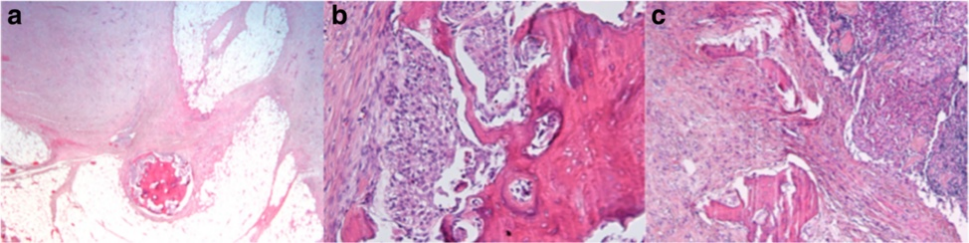
Histological findings at the time of the primary tumor resection (Hematoxylin & Eosin, H&E): The primary tumor consisted of a high grade urothelial carcinoma infiltrating the bladder wall; at the periphery of the tumor, a small focus of osteomedullary bone intimately admixed with the urothelial carcinoma was composed of small and non atypical osteocytes within mature-appearing trabeculae of lamellar bone (**a**, Original magnification 20× and **b**, 200×). **c**: a focus of bone metaplasia is seen at the hilum of a metastatic lymph node (100×)

A 75-year-old man was treated for a high-grade bladder urothelial carcinoma (pT3b N1) by endoscopic resection of the bladder with neo-adjuvant chemotherapy, followed by a right nephro-uretrectomy, a radical cysto-prostatectomy and bilateral ilio-obturator lymph node dissection. Macroscopic invasion of perivesical soft tissue was observed, and histology showed poorly differentiated carcinoma (Fig. 1a), with perineural invasion. On the deep leading edge of the tumor, small foci of osteomedullary bone metaplasia were observed (Fig. 1b). Non atypical osteocytes were seen within trabeculae of mature-appearing lamellar bone. Two out of seven lymph nodes were metastatic, among which one showed a focus of parahilar bone metaplasia (Fig. 1c). Reactivity to p63 is expected in 81-92 % of high grade urothelial carcinomas [5] accordingly both the primary tumor cells and a lymph node metastasis showed reactivity to squamous-associated marker p63 (clone 7JUL, mouse monoclonal, Novocastra).

**Fig. 2:**
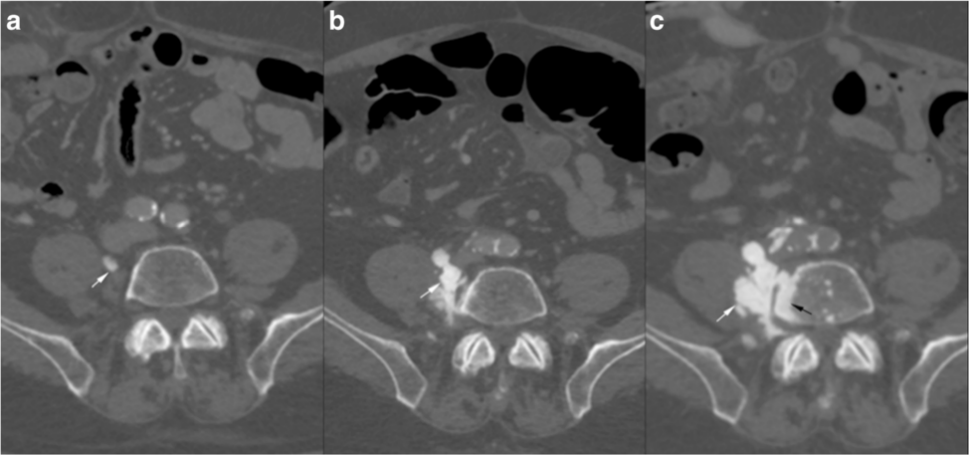
Follow-up CT performed 1 year after surgery (**a**) shows a small calcified nodule (white arrow) in the right retroperitoneal space. 6 months later (**b**) the calcified mass has increased in size. On another control 6 (**c**) months later the ossification progressed in size and into the adjacent vertebra (black arrow)

**Fig. 3:**
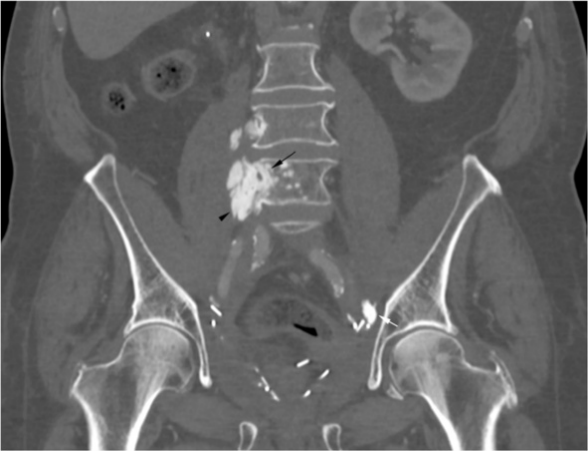
Coronal reformat of a CT shows the multifocal ossifications along the retroperitoneal space (black arrowheads) as well as the intravertebral extension (black arrow). A calcified nodule is seen in the left inguinal area (white arrow)

**Fig. 4:**
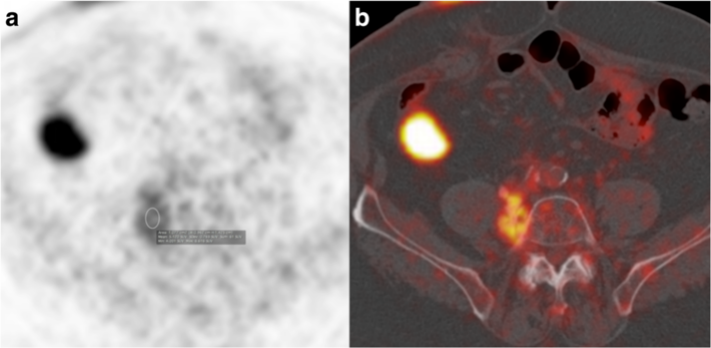
PET (**a**) and PET-CT fused axial slices (**b**) demonstrate the moderately hypermetabolic (SUV max 5.1) lesion of the retroperitoneum

Treatment was completed by adjuvant chemotherapy and radiotherapy for positive lymph nodes. Two years later, during normally scheduled imaging follow up, the apparition of several osseous formations localized in the right retroperitoneal space within the psoas muscle at the level of the bodies of 4th and 5th lumbar vertebra was noted (Fig. 2). The formations were very dense on CT (like cortical bone), lobulated and in continuity with normal bone with a parosteal location (Fig. 3). The largest mass measured initially 4 cm. A PET-CT (Fig. 4) showed that the mass situated in the right lower quadrant was moderately hypermetabolic with a max SUV of 5.1.

**Fig. 5:**
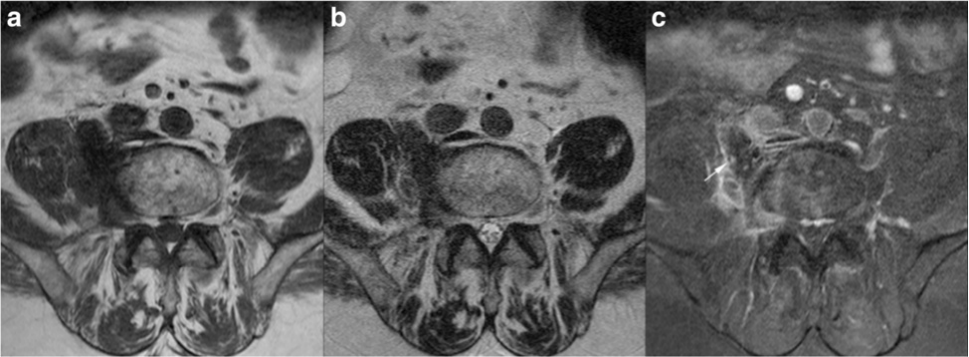
Axial MRI shows the lesions as hypointense on T2 (**a**) and T1 (**b**), with peripheral enhancement (arrow) on T1 fat sat after injection of contrast (**c**)

**Fig. 6:**
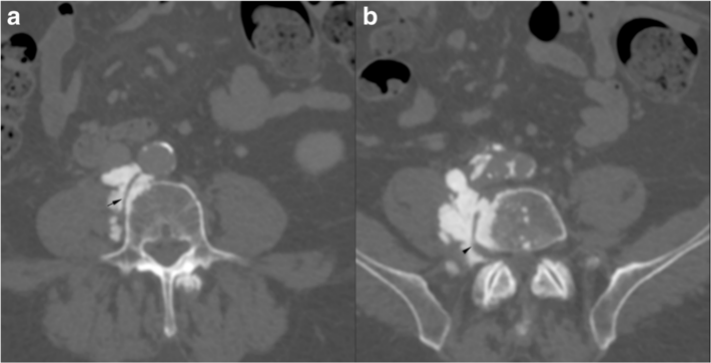
Axial CT slices (**a**, **b**) depict the sheating of the vessels by the ossified mass (arrow) as well as the sheating of the lumbar nerves (arrowheads)

**Fig. 7:**
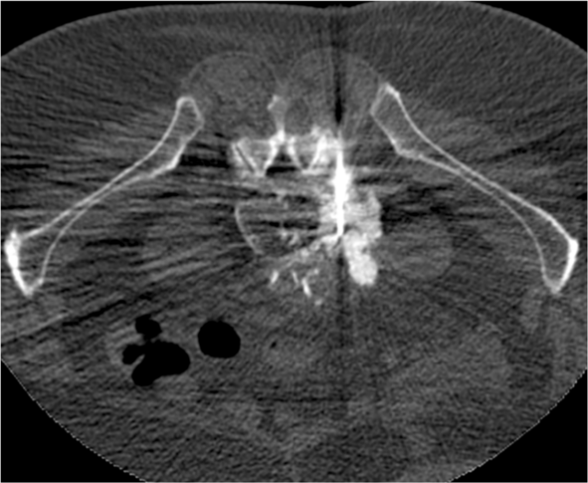
A percutaneous biopsy was performed under CT guidance using a Bonopty 16G biopsy set

A complementary MRI (Fig. 5) was performed and the lesions appeared hypointense on all sequences with thin peripheral enhancement. The multidisciplinary discussion recommended a control CT after 3 months. At control the osseous formations increased in size with vessel sheathing, including the inferior vena cava, lumbar arteries and also the 5th right spinal ganglia without extension in the foramina (Fig. 6). In addition, it showed the development of further foci of bone formation in pelvic ganglia sites bilaterally. Considering the rapid progression of the disease and the absence of a clear diagnosis, a percutaneous biopsy was scheduled. The procedure was performed under CT guidance (VCT,GE healthcare, Milwaukee, Wis) with anaesthetic stand-by using a Bonopty 16G/15 cm needle (AprioMed/Bonopty Bone Biopsy System) (Fig. 7).

**Fig. 8:**
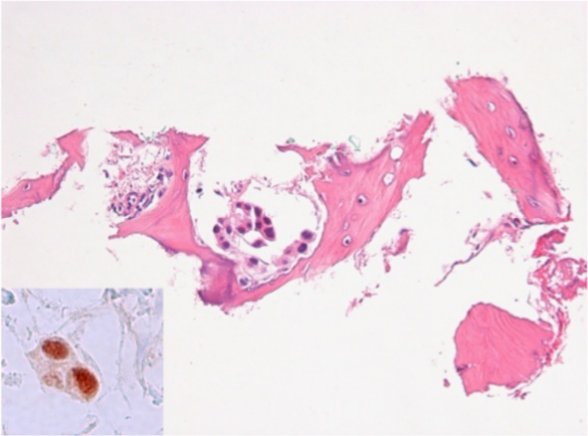
Percutaneous biopsy of the retroperitoneal mass showed non atypical lamellar bone trabeculae (H&E, 400×) associated with p63 positive malignant epithelial cells consistent with metastatic urothelial carcinoma (Inset, p63, 600×)

Histological study of the biopsy showed mainly crush artifacts, but also minute foci of lamellar bone trabeculae associated with broad spectrum keratin (clones AE1/AE3, mouse monoclonal, Dako) and p63 positive epithelial cells consistent with metastatic urothelial carcinoma (Fig. 8). Again, the bone trabeculae displayed no atypical features.

At the time of this report, the patient is still alive and only the mentioned lesions are progressing, without response to additional radiotherapy.

## Discussion

Bone formation in the stroma of bladder carcinoma or in heterotopic sites has been noted in a few reports [6–8]. 17 cases were reported in a review of unusual bladder carcinoma, published in 1991. These bone formation concerned the primitive tumor and in the majority of the reported cases, the tissue was benign-appearing [2, 3, 6]. The degree of calcification is reported from minimal to complete as in our case with a stony feature. Clones of tumour cells may induce osteogenesis leading to osseous development as transitional epithelium is a well-known bone-inducing agent [9]. However, bone production by tumor cells would indicate osteosarcomatous differentiation. The main hypothesis would be a transformation of undifferentiated stromal mesenchymal cells into osteoblasts [10]. Bone morphogenetic proteins (BMP) are known to induce primary new bone formation at heterotopic, extraosseous sites [11]. In particular, involvement of bone morphogenetic protein 2 (BMP-2) produced by tumor cells [12] in both primary and metastatic sites as well as the expression of a BMP type 1 receptor called BMP-Ib in the metastatic sites, were all reported to play a role in the osseous formation. (BMP 2) is admitted as an active inducer of osteoblastic metaplasia in primary site and BMP-Ib as a precursor in metastatic site. They act as cytokine, target multipotent cells and induce differentiation of mesenchymatal cells onto osteoblasts [10]. That heterotopic ossification might be an indicator of aggressiveness of tumor without characteristic morphological features of carcinosarcoma or primary osteosarcoma (absence of mesenchymatal cell proliferation, stromal cell mitosis and lack of sarcomatoid elements) [4]. The characteristics of this entity are not fully described nor understood. The degree of invasion, the course of the disease, the optimal treatment and the prognosis parameters are not known. These parameters should be further determined [13].

To our knowledge, this is the first reported case focusing on the aggressiveness of these metastases illustrated by a multimodality imaging approach. CT represents the best imaging procedure, and shows multiple osseous formations, located in the retroperitoneal space following lymph nodes sites, sheathing adjacent vessels, that may encase nerves and spinal ganglia, as in our case. Another finding was the invasion of adjacent bones with the same matrix. Furthermore, CT allows follow up of the lesions and detection of new lesions. The metabolic activity recorded on the PET-CT was moderate and the imaging pattern on MRI was non-specific, the osseous lesions appearing hypointense in all the sequences. The diffusion sequence does not contribute to the characterization of the lesion matrix due to the pronounced hypointensity on T2 sequences.

The case presented here showed rapid progression of the disease with risk of local compression; no vascular erosion or fracture was noted during the follow up period of 2 years and a half. This osseous metaplasia leads to the differential diagnosis of bone formation. In the context of carcinoma treated with radiotherapy and if the disease appears in the irradiation field, a radiation induced sarcoma should be the first suspected diagnosis. It is a well-known entity and the major criteria for the definition, controversial in the literature, is the length between radiation exposure and tumor formation: it seems that a latency of 6 months is sufficient to affirm the diagnosis in some cases [14]. Extraskeletekal osteosarcoma is more common after radiation therapy than spontaneous formation, and represents 1-2 % of all soft tissue sarcoma and 4 % of all osteosarcomas; the retroperitoneal space is involved in 17 % of cases. It occurs after 40 years of age. Prognosis is poor with a 5-years survival of about 11 % [15, 16]. Imaging studies show variable amounts of ossification in the tumor [17].

These areas are heterogeneous with focal hypoattenuation on CT and intermediate signal on T1 with enhancement [16] or low signal both in T1 and T2 sequences [18]. Areas with low attenuation are visible due to necrosis or hemorrhage. Local invasion can be encountered.

Malignant bone formation can be a feature of the sarcomatoid variant of infiltrating urothelial carcinoma, a well recognised entity [19], considered to be aggressive and of poor prognosis. Malignant heterologous elements can be observed in sarcomatous carcinomas of various organs, including urinary bladder; the malignant mesenchymal elements are produced by the carcinoma cells. However, no atypical features were observed in the heterotopic bone foci, both in the primary tumor and in the lymph node and retroperitonel metastases. The histological criteria of osteosarcoma were thus not met. From a contrasting point of view, osteoblastic bone metastases consist of benign bone trabeculae produced by non malignant osteoblasts under the influence of cytokines secreted by the carcinoma cells, an example typical for bone metastases of adenocarcinoma of the prostate. However, not one of the observed bone foci was in an intraosseous localization. Therefore, we believe bone formation in the case presented here represents metaplasia, defined by the production of benign elements in unusual places. Ossifying metaplasia thus refers to the production of reactive benign bone trabeculae. As the mass was osseous, a myositis ossificans and a melorheostosis should also be discussed.

Myositis ossificans is a benign solitary soft tissue ossifying mass in skeletal muscle, with a history of traumatism. The pattern of peripheral ossification is important to establish the diagnosis; mature forms may present a complete ossification [20]. The lesion is intramuscular, predominantly localized at sites of injury, and can occur almost anywhere. The CT gives a specific pattern with a well limited high attenuating periphery and a low attenuating central portion. On MRI, it is isointense in T1, hyperintense in T2 with enhancement after injection [21].

Melorheostosis is defined as a rare sclerosing bone dysplasia with a characteristic linear sclerosis of cortical bone; calcification and ossification of soft tissues are typically found near the large joints. It appears as low signal intensity in all MR sequences but intermediate signal is described with enhancement in soft tissue masses [22, 23]. Computed tomography demonstrates a well matured ossified mass. Bone scintigraphy shows moderately uptake of tracer.

Tumoral calcinosis can also be discussed in the presence of a massively calcified mass [24]. It is a rare disease, a complication of chronic renal failure. The main features are the lobulated aspect and the periarticular location. It affects young adults and appears in imaging exams as a heterogeneous dense mass without bone involvement.

Amyloidosis was also considered in the differential diagnosis. It is a heterogeneous group of disorders and in systemic form, amyloid may infiltrate the retroperitoneal and pelvic soft tissues and undergo gradually progressive calcification over time with formation of soft tissue thickening, encasing the urinary tract and involving renal sinus. CT shows amorphous calcification associated with tissue thickening, MRI demonstrates intermediate signal intensity on T1 and decreased signal intensity on T2 [25].

Other rare, benign neoplastic causes of calcified masses in the pelvic retroperitoneum include ganglioneuroma, schwannoma, paraganglioma, hemangioma, and presacral teratoma [26].

## Conclusion

To the best of our knowledge, this is the first reported case of retroperitoneal osseous metaplasia in a urothelial carcinoma metastasis studied with different imaging modalities and confirmed with percutaneous, CT guided biopsy. In this case we also demonstrated the aggressiveness of the disease, the rapid progression and the sheathing characteristic of adjacent organs including vessels and nerves. Sarcomatous carcinoma and radiation induced osteosarcoma are the most challenging differential diagnoses even though delay was short.

Prognosis is likely determined by the extent of the high grade urothelial carcinoma, but independent of the benign metaplastic bone component.

## Consent

Written informed consent was obtained from the patient for publication of this case report and any accompanying images.
